# A Combination of Action Research and Reflective Journal Writing in an English as a Foreign Language Class: Learners’ Psychological Point of Views and Their Grammar Use in Writing

**DOI:** 10.3389/fpsyg.2022.810775

**Published:** 2022-05-16

**Authors:** Soheila Tahmasbi, Shabnam Karimnia, Ali Rahimi

**Affiliations:** ^1^Department of English Language Teaching, Abadan Branch, Islamic Azad University, Abadan Branch, Abadan, Iran; ^2^Young Researchers and Elite Club, Islamic Azad University, Abadan Branch, Abadan, Iran; ^3^School of Social Sciences and Languages, Vellore Institute of Technology (VIT) University, Vellore, India

**Keywords:** reflective thinking, reflective journals, grammar of writing, action research, EFL classroom

## Abstract

Action research (AR) and reflective thinking (RT) can enhance learning since both processes provide students with the opportunities to step back and think about how they actually solve problems. While there is a robust academic inquiry on reflection practices and AR in the educational setting, investigating learners’ reflections through AR practices can shed more light on related research. This study implemented reflective journal writing through AR and aimed to investigate (1) the participants’ views about reflective journal writing, (2) the effects of journal writing on RT development, and (3) the learners’ grammar use in writing. Eighty language learners formed the two experimental and control groups of the study. The possible relationship between the RT level and participants’ final exam was checked. Analyses of the participants’ journals, the semi-structured interview, the questionnaires’ results, and the final exam scores were considered. The findings showed that the participants had positive views about journal writing, and they could enhance their level of RT as well as their grammar use in writing; nevertheless, no relationship between the RT level and final exam scores of the participants was found. The methodology and the results of the study could be conducive to welcoming alternative methods of teaching and assessment that encourage the learners’ reflective practices and active engagements in language classes.

## Introduction

In daily routines, people think about their past actions, explore their experiences, and evaluate their thoughts and feelings. If individuals are deliberately engaged in such intellectual and affective activities to enhance their knowledge, obtain promising results, and reach a better understanding, the process is called reflection (Pretorius and Ford, 2016). Reflection as an ‘active, persistent, and careful consideration of any belief or possible image of knowledge’ (Dewey, 1933) also describes deliberations. Through deliberate activities, individuals step back and analyze their experiences or feelings (Schon, 1983) to finalize better goals (Costa and Kallick, 2008; Ramlal and Augustin, 2020). Reflecting is both an attempt that makes sense of the experiences and a potential that can end in personal transformation for the individuals involved (Van Velzen, 2017). Complex introspective and metacognitive processes of reflective practices are connected to critical thinking and self-assessment. Some reflective practices can foster individuals’ reflective thinking (RT) and linguistic skills (Tavakoli and Davoudi, 2016; Ramlal and Augustin, 2020).

How individuals develop intellectual and affective activities to reach new understandings and appreciations has been investigated in educational settings (Costa and Kallick, 2008; Turhan and Kirkgoz, 2018). Whenever students are instructed to reflect in practice, they will be actively involved in reflective practices (Barnett and O’Mahony, 2006); fostering the students’ capacity for reflective learning is part of fostering their “capacity to learn how to learn” (Bourner, 2003). Reflective practices are welcomed in education because these practices help students recognize their learning needs and contemplate their own values and beliefs. It is possible that students even extend reflective practices to their learning and future qualifications (Mann et al., 2007). Reflection has also been a crucial component to enhance intellectual growth and has shown support to thinking and doing of students (Rampersad and Herbert, 2005; Ramlal and Augustin, 2020).

Despite the merits mentioned, it is noted that the path to reflection may not be a smooth one (Phan, 2008; Moradkhani and Shirazizadeh, 2017; Soodmand Afshar and Farahani, 2018). van Velzen (2015) emphasized that learners might face some barriers toward reflecting on how they learn something and this makes them reluctant to reflect. It is even argued that teaching learners to reflect seems tough (Rogers, 2001). In the same vein, learners may resist reflective practices since the process can insert mental (van Velzen, 2015) and psychological (Phan, 2008) demands for the individuals. More recently, studies conducted in the context of the present study focused on the impediments of reflective practices from the perspectives of Iranian English as a foreign language (EFL) teachers. Soodmand Afshar and Farahani (2018) classified different inhibitors in their study. They reported affective, emotional, cognitive, and learning situation inhibitors as factors that their teacher participants believed to negatively affect the students’ reflective practice.

So, how can learners reflect on their own learning process to benefit from reflections while hurdles are around? How can teachers enhance common teaching practices and effectively go beyond course mastery to foster the learners’ reflective practices? How can instructors engage learners in reflective practices during the courses and possibly extend reflections to the individuals’ future professions? (Demir, 2015). Although it is critical to answer these questions, the characteristics of the Iranian EFL context negatively affect answering these questions. Students are raised to be obedient, and knowledge-receivers and teachers are trained to comply with authorities and prescribed practices (Tahmasebi and Yamini, 2013). In fact, classroom practices in which students are encouraged to reflect on their own learning are rare, if not absent. Concerns are also confined to research endeavors where interested teachers try to bring changes in the dominant learning/teaching trend (Soodmand Afshar and Farahani, 2018). As Avgitidou (2020) states, the verbal lecture is still almost the dominant teaching method in some countries. Although it would be too optimistic to invite teachers and learners to make decisions about the content and process of education, it is possible to try learning situations where negotiation and reflection over learning is part of the process. Developing reflective practices like journal writing through AR (Frazier and Eick, 2015; Davis et al., 2018) can be a possibility for teachers to relegate part of the teaching processes to learners and diminish the teachers’ roles as knowledge transmitters and change students’ roles as mere obedient agents.

Action research shares common grounds with reflective practices in the related literature, and it seems encouraging if we use them in a single study. Webb and Scoular (2011) believe that the process of AR involves cycles, the first of which is a reflection on the practice and identifying the problem. Acting, experimenting, observing, reflecting, analyzing, and evaluating are other phases. These cycles share common grounds with reflective practices. Messiou (2018) found that teacher collaboration and reflective practice would lead to actions. Furthermore, teachers’ support increases the active participation of students for further reflection and changes in learning practices. In her study, she exemplifies a teacher who made further changes in the learning process. In the teaching and learning process *via* the instructor-learners’ reciprocal relationship, reflection can be enhanced and can positively affect the two parties. Forester et al. (2019) believe that AR is beneficial in two ways. First, it helps participants account together for what they know and how they know something, and second, it has a transformative role in which participants change their thoughts about values and what ethically matters. That is, students involved themselves in actions in order to be desirable for their contexts. For Cook (2004), the success of any learning practice is connected to how learners apply that learning in the future. She states that learning is not just the result of what we do but the result of how our students give meaning to what they have learned also matters.

These highlights and the vacancy in aligning AR and reflective practices in the literature led to the present study. This study aligned AR and RT as focal processes in promoting learning. As some researchers have argued, teachers have the autonomy to make pedagogical decisions based on the investigation of their own practices (Whitehead and McNiff, 2006; Abednia et al., 2013; Rossi and Thorsen, 2019). We also followed this view and considered teachers as reflective practitioners who can implement AR to enhance the reflection in their learners’ actions. How learners’ reflective levels may benefit from their reflective journal writing when AR cycles are implemented (by the teacher) and how individuals view their practice of reflective journaling are issues that the present study aims to investigate. Taking one step beyond the reflective component of journaling, relatively little is known about the learners’ kind of sentences in journal writing. It should be noted that reflective journals can be dual assets through which learners’ language development can be monitored and their reflections over a particular course experience can be considered. The purpose of the present study is to first assess the learners’ RT levels, evaluate whether there is evidence of reflection in student-written journals, investigate whether students show improvements in their reflective skills through journal keeping. Furthermore, the study also aims to analyze students’ journals by subjecting their reflection journals to text analysis. A brief literature of reflective journal writing, AR, and the place of grammar in language learning, results, discussion, and conclusion are the following sections of the present study.

## What Is a Reflective Journal?

Reflective journals are defined as written entries that students develop when they think about what they have learned. Students report on past processes involved in their learning or conversations that happened between students and teachers during a particular time. These practices help students gain insights into their own development (Thorpe, 2004; Lew and Schmidt, 2011). Reflective journaling also includes self-reflection and learning strategies through which learners improve their reflective capacity and skills. These reflective tools may consequently aid students to organize the knowledge they have practiced, get rid of negative feelings, and be responsible for their own learning (Carey et al., 2017; Zulfikar and Mujiburrahman, 2018). For Lew and Schmidt (2011), the reflection component of journal writing is the attraction point in the learning context.

Despite the prescribed roles that most Iranian learners have grown up with, research shows that they welcome alternative activities when they are asked by their teachers (Abednia et al., 2013). For the participants and the teacher of the present study, classroom time and content were almost planned by the head branch of the institute centered in Tehran. In such language classes, students’ journals seemed a convenient method to develop reflections since learners carry on language learning activities at home; and the instructor could assess the journals and provide feedback outside the classroom time.

For Hubbs and Brand (2010), asking about assessing a reflective journal is a “thorny” question. They argue that journal assessment depends on the course type, learning outcomes, and the context of learning and suggest a “comprehensive” assessment of reflection. However, other researchers (Stewart and Richardson, 2000) recommended reviewing the context of the reflective journal instead of assessing it. In this piece of research, guided by the nature of the research questions and previous similar studies, the contents of the participants’ reflective journals were analyzed. A content analysis of journals provided the present authors with possible traces of the participants’ reflection, improvement, and grammar use in their journals.

The learners’ need for grammar lessons in EFL classes is one of the most crucial concerns of teachers and learners. Most of the time, it is learners’ grammatical knowledge that is assessed through final multiple-choice items that determine their language ability (Zhou et al., 2014). Similar concerns about language learners’ grammar are common in the Iranian language learning classes. Avarzamani and Farahian (2019) believe that the Iranian instructional system somehow follows the traditional approach of product-oriented writing. They argue that many teachers in the existing system think grammar and vocabulary training would be enough for writing instructions. These policies diminish the prominent role of processes in EFL writing courses. This has led to a lack or little awareness of the cognitive complexities in the processes of constructing a text, particularly in the foreign language (FL) contexts.

Cannady and Gallo (2016) found that, when writing assignments focus on higher-order cognitive skill development, active learning is enhanced. According to Bloom (1956), particular activities end in particular types of learning. He developed the original categories of knowledge, comprehension, application, analysis, synthesis, and evaluation as taxonomy levels of cognitive skills. At the first level, the knowledge/remembering level, just memorization, and recall are involved and the lower order cognitive skills are engaged; hence, memorized information is not internalized. However, at the upper levels of Bloom’s taxonomy, more critical thinking occurs. Baghaei et al. (2020) argue that the highest dimension involves combining the parts together so that a creative whole is built. Whenever writing leads to higher-order cognitive skills, it becomes a helpful asset to facilitate learning and develop critical thinking (Ryan, 2013).

Overall, in this research, reflective journals include the experimental group’s reflections on the lessons of their integrated language course in a language institute in Iran. Students expressed their views about the content of the lessons they received, and their teacher provided feedback to their concerns. Overall, we considered the students’ reflective journals to explore new understandings about the learners’ reflections on their own learning and their grammar use in journals.

## Action Research

As a reflective process, AR includes inquiry and discussion as components of research (Ferrance, 2000; Suzuki et al., 2009). Researchers and practitioners work together to apply theory, open new windows toward a theory-practice connection, and achieve language improvements (Reason, 2006; Carter, 2012). AR reflects both problems and potential in schools and classroom contexts (James and Augustin, 2018).

As AR is generally done in small scales and particular contexts, its validity is questioned (McDonough, 2006). In fact, AR generally does not test hypotheses, manipulate variables, or generalize findings. That is why there have been speculations with regard to the question of whether AR can be considered as “real research.” McDonough (2006) argues if one evaluates AR based on the validity criteria related to qualitative research, the validity criteria for practitioner research, and the TESOL Quarterly (2003) guidelines for case study research, and the validity questions of AR are all answered.

Similarly, AR will not answer all concerns about how students learn or what educators may do to improve practice, but it is carried out in a particular place where there are concerns to bridge the distance between theory and practice and give students more prominent roles (Ferrance, 2000; Zulfikar and Mujiburrahman, 2018). In the Iranian educational settings, teachers are almost expected to teach book contents and implement predetermined syllabus and materials (Avarzamani and Farahian, 2019); hence, AR, as well as reflective practices, can effectively raise the teachers’ and learners’ understanding about the process of teaching and learning and possibly provide a more intimate context for language classes. In this study, we hypothesized that if teachers are involved in the stages of making plans for actions and implementing planned actions, they can have an impact on the learning and teaching process. As such, teachers are not just the classroom authority, that is, as researchers we have addressed concerns over which we might have some influence.

In the related literature, AR in education is encouraged for activating both students and teachers to continuously develop themselves (Edwards and Burns, 2016) through teacher-students authentic interactions. Following this view, the authors of the present study hypothesized that integrating an AR method into a journal writing process may bring a change in the immediate language learning classes. It is also argued that, if teachers and learners have more connections, the learners’ reflection practice would possibly improve their RT levels and their grammar use in writing. In most educational settings, these improvements are in line with course objectives. In other words, the present authors followed Kemmis and McTaggart (1988) and implemented a four-cycle AR. Language learners developed their reflective journals and presented their reflections. Throughout the stages, it was aimed to investigate how participants of the present study, who were language learners of an Iranian institute, view their own learning of course materials (such as reading passages of the coursebook, and question making).

Among different models, the earlier cyclical structure of AR presented by Kemmis and McTaggart (1988) includes the four stages of plan, act, observe, and reflect. This model can be implemented into classrooms and can include common classroom teaching practices (Richards, 2003; Edwards and Burns, 2016). In brief, these stages are:

Plan: develop a plan of critically informed action to improve what is already happening.
Act: act to implement the plan.
Observe: observe the effects of the critically informed action in the context in which it occurs.
Reflect: reflect on these effects as the basis for further planning, subsequent critically informed action, and so on, through a succession of stages (Edwards and Burns, 2016).

The guiding research questions were as follows:

1. How do participants of the study view reflective journal writings? How do the journal writing practices affect the participants’ level of RT?
2. To what extent does reflective journal writing affect learners’ grammar use in writing?
3. How do learners’ RT levels affect their achievement scores?

## Methodology

The present study aimed to investigate participants’ views about reflective journal writing and check the possible effects of journal writing on RT development and grammar of writing. A four-stage and cyclical AR was followed.

### Participant

The participants were Iranian EFL learners in a language institute in Abadan, Iran. The institute has branches in all provinces all over the country; hence, language level, course content, sourcebooks, and even final exams are determined by the head branch, which is in Tehran, Iran.

First, two intermediate classes, each holding 40 female learners ranging from 15 to 32 years of age were included as the experimental and control groups. As the age range shows, the institute placed participants aged from 15 to 36 according to their language levels. As the age of the participants points out, some of the participants were high school and college students (32 people), and the rest were employees filling ranks in the offices as clerks or doctors, so they had work experience besides class life experience. Intermediate students were considered because they could understand related terminology and they had stronger control over the organization of language in their writing.

Based on institute regulations, such an intermediate course should run for 20 sessions with a focus on developing four skills: speaking, listening, reading, and writing. In fact, the institute places language learners into six levels: basic, elementary, pre-intermediate, intermediate, high-intermediate, and advanced. After the initial registration in the institute, students are placed in different classes based on a multiple-choice placement test prepared by the central assessment department, and then, test interviews were run by some teachers of the institutes. Our participants were able to make wh-questions as well as yes/no questions. Moreover, they have practiced paragraph writing and other skills like speaking/listening and reading English.

## Research Instruments

The following instruments were used at different stages of the study.

### Reflective thinking questionnaire

Once at the onset of the study and once after journal writing, Kember et al.’s (2000) 16-item reflective thinking questionnaire was used. It was used to identify the participants’ reflective thinking level.

The questionnaire was used to identify the participants’ RT level.

### Reflective Thinking Journal

The participants were asked to record their reflections on their class life including their experience on the language learning course, question-making practices, and the taught passages during the study.

### Reflective journal assessment coding

Kember et al.’s (2008) four-category coding for reflective journal assessment was used for evaluating and reviewing participants’ journals. The coding scheme included the same four categories that Kember et al. (2000) used for determining levels of reflection in their reflective thinking questionnaire. The scheme included four levels: (1) habitual action/non-reflection; (2) understanding; (3) reflection; and (4) critical reflection.

### An Overall Class Achievement Test

It was used to compare the performance of the two groups of participants at the end of the study. It was a 60-item multiple-choice final exam including four parts: listening, vocabulary, grammar, and reading prepared by the institute assessment committee with a total score of 100. In fact, the performance of both groups on this final exam was part of their course requirement.

### Bloom’s Taxonomy

It is a framework suggested by Bloom (1956). The taxonomy is composed of six classifications: knowledge, comprehension, application, analysis, synthesis, and evaluation. Participants’ journal structures were classified based on Bloom’s (1956) Taxonomy to determine the learners’ thinking level.

### A Semi-Structured Interview

It was formed one week after the end of the course. In the interview, the participants were asked the following:

To talk about their experience of reflective journal writing, and to reflect on journal writing as a form of class activity.

If they faced any challenges while keeping journals, what solution they would suggest for the problems, what solution they had possibly experienced in the process of journal writing, and if they reflected even after the course.

### Procedures

The study was structured over almost 6 months (it included three final sessions of Intermediate I, and all sessions of Intermediate II. Although participation in the program was voluntary, all learners participated).

The principal concern was to develop the participants’ understanding of reflective journaling involved. In a 90-min session, the concepts were introduced and some samples of written journals were presented to the participants. The learners were asked to keep a reflective journal to record their reflection on class life including problems regarding question making and reading passages. They were told that the activity of journal writing was not part of their course requirements, and their journals will not affect the final exam and course grades. Some learners preferred to make hand-made, well-designed notebooks as their journals while some bought a small notebook. Actually, the first page of all the journals was the same: the presented definition of reflection and RT were jotted down. Afterward, every session, while conducting AR cycles, the learners had proper time to write their reflection on their learning process, question-making practices, and taught passages. To make sure they understood the concept, they were asked to write their reflections on the overall course in English (in Intermediate I) for two sessions. They were asked to reflect on what they mastered based on book texts, grammar drills, and even the whole class life experience during that semester. Next, the instructor observed the process of journaling in the classroom, and the cycles were repeated whenever necessary. The instructor and the learners discussed the reflections individually or in groups. Meanwhile, the participants in the control group did not develop journals and did not follow the cycles of AR. They shared the teacher, the coursebook, the final exam, and even all other class activities with the experimental group. The study was carried out in an institute that is affiliated with the main institute (which has branches in all provinces throughout the country). All the branches of the institute follow even the same teaching methods and procedures in their language classes. The control group also received the practices and followed the same procedures common to all other classes including the experimental group.

The students attended their classes 2 days a week. Every session, the instructor reminded the participants to write down their reflections about the class activities and their learning after the class. The activity of journal writing was done at home. They were told they can write their reflections in notebooks or diaries. It is said no guidelines should be given regarding the amount that is written under each heading or journal topic because it would be over-structuring (see Hubbs and Brand, 2010). However, students were encouraged to provide regular journal entries for each class activity and learning. Their journals were gathered just once a week (every two sessions), and the writers were provided with some feedback.

After that, the possible contribution of reflective writing to the learners’ grammar use in writing was analyzed since as it went on, writing is an embodiment of correct grammar. To prevent particular test-effect factors, they were not told that their grammar of writing will be considered. The actions so far were implemented during Intermediate I in the fall semester.

It should be mentioned that, although learners got familiar with the concepts of reflection and journal writing and were told about their involvement in the study, they were not aware of the AR cycles, and the teacher implemented the cycles. The main implementation was planned for the winter semester, which ran from January to March, and included a review of previous concepts, practices, acting, observing, data collection, data analysis, and reflection:

1. The experimental group participants and the instructor reviewed what they planned to develop, i.e., a reflective journal. This phase was not as easy as it comes first. A couple of learners, although volunteered to participate in the project, doubted if such practices could negatively affect their learning. Such concerns may further imply if reflection practices were not what participants are used to in their language classes. It is also said there is a vacancy of critical movements to enhance our deep understanding. As teachers, we should improve our wisdom about teaching and learning and develop our ability to recognize the limitations (Widdowson, 2003). The students were told that journal writing was not part of their course requirement. In fact, students would not receive grades from journal writing as an assignment or lose any grade if they would not write journals. Participation was voluntary; students could stop journal writing and leave the study whenever they wanted.
2. The participants in the experimental group developed their reflective journals. They included their reflections on course passages, the context, the grammar drills, and the class procedure in their journals. In fact, participants had enough time to develop reflective journals as a take-home activity regarding the what, how, and why of the lessons.
3. In line with the observation cycle, every two sessions, two of the researchers examined the reflective journals to check the class reflections and gave feedback either to the whole class or to the individuals either through writing notes or orally. Interestingly, the learners clarified their reflection by drawing some stick figures and simple shapes to show their emotions.
4. In the third cycle, participants’ journals were gathered to be evaluated. The researchers analyzed the content of the journals and assessed reflected statements.
  • Next, regarding the quantitative-qualitative design of the study, to measure the participants’ RT level, Kember et al.’s (1999) sixteen-item RT questionnaire was used. Although the overall content and order of the items did not change, it was required to include minor changes. For example, words like lecture and reappraise were replaced by more familiar words like teacher and evaluate. Although it is argued that this questionnaire is reliable and workable since it can measure reflection as an awareness and attitude with closed-ended, self-report, and rating scale questions (van Velzen, 2015), the questionnaire content was adapted and some minor changes regarding the content were done; for example, instead of course content, we included the name of their own course. Moreover, to make sure that the language knowledge of the students did not affect their performance on the questionnaire, the Persian meaning of the statements appeared beneath each item. Herein, the researcher read all the 16 items of the questionnaire out to the learners and clarified the technical or difficult terms and the participants ticked their answers (A) definitely agree, (B) agree only with reservation, (C) a definite answer is not possible, (D) disagree with reservation, and (E) definitely disagree.
  • This questionnaire was once implemented before conducting AR (before starting journal writing practices) to check the possible changes of the participants’ RT level. Kember et al.’s (2000) questionnaire was adapted for the local context for a similar group of intermediate level learners of the same gender, the previous term which assured the instructor about the reliability of the questionnaire.
  • After the second implementation of the questionnaire, some prompting questions (Denton, 2018) were asked: What happened to you while journal writing? Discuss the event of reflective journal writing and the activities you possibly performed. How do you feel about the results? How did this assignment make you feel? What part did you like? What could you have done better? What have you learnt from this event? How will this practice be useful in or related to your future studies or career?
  • The journals were collected and utilized as data collection tools; the researchers assisted by an experienced colleague of the institute evaluated the journals. The kinds of sentences they were making in their journals, both about their learning process and possible problems, and the grammar of their sentences, either simple or complex, were considered. In their writings, some students found it useful to be able to express their emotions (e.g., frustration and stress) in their journal entries. They reported that their journal writing enabled them to defeat some negative feelings and concentrate better on the course material. They reflected on what they learned in that course (e.g., technical vocabulary related to reflection and AR) and what they expected to learn and have not yet mastered (fluency in speaking). In addition, the learners also explained their strengths, weaknesses, and experiences in the classroom. Every alternative session, after providing feedback to the participants, the instructor engaged learners in continuous collaborative dialogues. For evaluating and reviewing the journals, Kember et al.’s (2008) four-category coding was followed (Kember, 1999; see also Kember et al., 2008).

The coding scheme uses the same four categories for determining the levels of reflection in the questionnaire. The scheme included four levels: (1) habitual action/non-reflection; (2) understanding; (3) reflection; (4) critical reflection.
Non-reflective writing occurs when students look for the material and put it into their writing without thinking about the material. They do not try to shape a new idea. Plagiarized material, paraphrases, and summaries are included in this category. At the level of understanding, students write based on what is in the textbook or the teacher notes; they understand the theories. However, since there is no reflection, the students cannot provide examples of how the theory relates to a practical situation. Furthermore, they cannot connect concepts to their personal life. At the third level, reflection happens. The concepts are interpreted in relation to personal experiences. Writers consider new situations and discuss materials. At this level, personal insights move beyond books, theories, and teacher notes. The writers usually describe emotions caused by the experience and they can judge and predict their experiences. Although writers make comparisons, they do not make conclusion based on their comparisons. Finally, a piece of writing may show students’ critical reflection. When the writing shows some evidence of a change in perspective over a fundamental belief, critical reflection is present. Writers question their experiences and fallacies and reflect on the evidence of modifying their biases and thoughts.

Finding the initiative, learners’ reflections are more descriptive rather than evaluative. The authors attempted to clarify the process by showing them some exemplars of reflective journals (Hume, 2009; Tavakoli and Davoudi, 2016). At the end of the study, students were asked to answer the following questions (on the last page of their journals):

- How did you find the experience of RT journal writing?
- Do you still reflect?
- Did you face any challenges while keeping your journal? What was your solution?

The answers to these questions were considered as participants’ overall reflections on journal writing.

At the final stage of the present study, the performance of both experimental and controlled groups (the former was run through AR and practiced journal writing, while the latter followed institutional common practices) on the objective final exams was considered as an indicator of class achievement. Like other branches of the institute that are located all over the country, participants of the present study received a final exam at a scheduled time. The exams are developed by the head office located in Tehran. The exam was administered at the same time for all intermediate-level students throughout the country and addressed all the taught skills and points to be covered during the course. So, the likely relation between the RT level and class achievement could also be investigated. Finally, participants’ views toward journal writing were unanimously gathered. They were asked to check if the overall process of journal writing was something positive, negative, or indifferent for them.

## Data Analysis

First, participants’ views about journal writing were analyzed descriptively based on a three-level scale: positive, negative, and indifferent. Second, the content of the journal was analyzed based on Kember’s (1999) coding schemes. Third, the participants’ performance on the first and second implementation of the Reflective Thinking Questionnaire, and their final exam results were analyzed using SPSS software. *T*-tests and correlation tests were used for the analysis. The former was used to check the possible changes of participants’ performance on the two administrations of the Critical Thinking Questionnaire and the latter to check the correlation among their final exam results and RT levels. After that, the interview content was analyzed following the same coding scheme. The grammar of the journals was also considered regarding the structure that the participants used during their journal development. The criterion for assessing their grammar use was the structure of the sentences, e.g., simple structure (Subject + verb + object) or complex structure.

## Results

To find the answers to the three questions of the study, the following analysis results were obtained. For Question 1, findings related to participants’ views on reflective journals, the results of the semi-structured interview and the results of participants’ performance on the first and second administration of the RT questionnaires were considered. To answer Question 2, the analysis results of the sentence structure of the journal sentences were used. To answer question 3, participants’ class achievement results were considered.

### Participants’ Views on Reflective Journals

Overall, the reaction of participants toward the journal writing experience could be considered positive. In fact, deciding upon participants’ views about journal writing was based on what they presented at the end of their journals. About 90% of them directly expressed that they liked the experience and would prefer to have similar practices in the future, and the researchers considered their reflection as positive. That is, out of forty participants, 36 suggested positive views and believed journal writing was a positive experience as part of their learning; two people had indifferent ideas. Two participants also suggested negative ideas toward journal writing; they suggested that journal writing was more time-consuming than what they assumed and they could have spent their time on learning their course materials instead. However, these two also confirmed that journal writing was an opportunity to share their feelings with the teacher. In fact, it can be said that 90% of participants suggested that journal writing provided them the chance to share their insights with their teacher and receive feedback, thereby expanding the students’ knowledge of and insight into the reflection practices and the lesson, as well as fostering better relations with the teacher. Parnia, a university student, and Asa, a clerk, respectively, suggested:

‘I found journal writing fantastic! I learnt so much from the content of the passages. So far, I just tried to read the passages and answer questions and learn new vocabulary, but while writing journals I learned about myself. It was a good experience.’
‘Not only was journal writing a good tool for learning more about the passages and lessons, I found it great to practice my writing and talk about my understanding. I think other learners feel like me, they have learned a lot in this course. I hope journal writing remain as homework for all students at schools.’

As it went on, the analysis indicated that most students positively viewed reflective journals that enhanced their learning and seemingly talked about greater self-awareness. Reviewing the journals, the researchers found that the content of all journals for each session was almost shaped into three parts.

First, as participants were asked to reflect on their learning, they almost began by presenting an introductory paragraph about the passages and summarizing the text and question-making practices (e.g., ‘the passage was about travelling […]’). Regarding the mentioned coding scheme, this part of writing reflects the understanding level of the participants. The second paragraph of each entry in the journals was self-reflective (‘I think […]’; for me; as long as I know, …), and when expressing their reflections on each passage, they talked about the effects of the passages on their feelings or their thoughts (‘I like this passage because it teaches me how I can get more successful’; ‘after reading this passage I tried to do more physical exercises’); and sometimes they talked to the teacher (e.g., ‘you know, I was always interested in physics’). All these writings are included in the reflection level, and critical reflection was not evident.

Of all journal writers, 90% stated that they never kept a journal before. Only 10% had developed journals for school topics and even participants who were university students admitted that they had not yet practiced journal writing for university courses. Regarding the wide age range of the participants (15–36), the content of the journals also reflected different concerns. The younger ones who were high school and university students almost talked about their learning experiences and schools (‘I think in this session I wrote the questions more easily, and it is noteworthy for me’; ‘I remembered school exams, and I was full of fear’), while the older ones talked about their jobs and daily routines. Actually, the age difference among the participants resulted in different reflections. Such results may imply that reflection practices may be beneficial in different situations for different people.

The next paragraphs formed students’ reflections on the effects of journal writing on their learning. The participants described the emotions evoked by the experience of journal writing, and they even judged their learning experiences. They compared their past and present experiences of learning. However, they did not draw conclusion based on the comparison. One of the learners, for instance, suggested that:

Journal writing is something new for me, but it is a great way to help me reflect on what and why I have done something in the classroom. It is like looking at myself in the mirror. (Mary)

Mary’s insight about journal writing reveals that she has perceived the journal writing process as helpful to improve her learning and alertness but does not go further to provide critical reflection. Similarly, Tina, one of her classmates, wrote that she felt deeper understanding in her learning due to journal writing:

In my reflective journal, I could monitor my learning. Moreover, I could concentrate on my strengths and weaknesses. In this way, I caught the course content deeply. (Tina)

Critical reflection was evidenced in one of the written journals. One of the participants, Ana, mentioned, despite her hectic life, that she liked reflective journal writing since this action could influence her work life as a dentist. She said that she would ask her patients more detailed and clearer questions and appealingly she was faster at solving office problems.

In the present study, various figures or drawings have been drawn by the participants in the journals to show their reflection in their own way; it can be said participants did not always share a common way to reflect in the journals.

Despite this, two participants were not sure about the positive results of journal writing as they said they could not find enough time in their hectic life to jot down their learning process and they liked to search for alternative means.

### The Interview

To enhance the credibility of the research (Moradkhani and Shirazizadeh, 2017), fifteen participants out of forty of the experimental group were randomly selected for a semi-structured interview. Similar to what they were asked in journal writing, the learners were led to answer some questions:

- How did you find the experience of RT journal writing?
- Do you still reflect?
- Did you face any challenges while keeping your journal? What was your solution?

Then, the interviewees presented their insights toward reflective journal writing. In this way, the effectiveness of reflective journals as a practical tool to watch the learning progress and boost awareness was reported in some of the participants’ narrations. The interviews were recorded and then received content analysis.

Participants’ answers were classified based on the questions raised by the interviewer (their course teacher). First, they admitted that they found the process of journal writing useful for their learning. Some believed that journal writing as a take-home assignment can enhance their reflection and writing skills, e.g., Jane, a bank clerk, believed ‘while writing I looked for proper words and tried to write correct sentences and I practiced more sentences.’ As it went on, they stated that they even reflected on their daily activities and decisions. However, they said that journal writing was challenging for them because they had to spend more time for their language course and because they had to check the words and grammar and they could address this problem by predicting the results, which was getting more from the course.

All in all, all interviewed participants suggested that the course has made them “feel differently” in the classroom. Moreover, they added that they became more conscious of various changes and improvements while studying. Eleven participants who were clerks believed that they even “felt differently” at work by more quickly asking sharp and detailed questions or solving the office problems thoughtfully. For example, Kate, a teacher, asserted that, when she was teaching, she could feel the effects of reflective practices, and she practiced reflections in all teaching phases.

To summarize this section, learners’ views on reflective journals and interview results were connected to question number one of the study, which asked about the participants’ views on their reflective journaling. The analysis results of journals’ sentences and participants’ performance on their final achievement tests provided answers to questions 2 and 3, respectively.

To further consider how journal writing practices may affect the RT level of the participants, both groups’ (those who created journals and those who did not) scores on the first and second administration of the questionnaire were compared (Tables 1–4).

**TABLE 1:** Descriptive statistics of both groups in the first administration of the reflective questionnaire.

Groups	*N*	Mean	SD	SEM
Experimental	40	56.27	4.78	0.75
Control	40	53.37	4.66	0.73

**TABLE 2:** Paired samples *t*-test results on the experimental group’s reflective thinking level.

	Mean	SD	SEM	*t*	*df*	sig.
Experimental Q1–Experimental Q2	−3.27	4.6	0.73	−4.47	39	0.000

**TABLE 3:** Paired samples *t*-test results of the control group’s reflective thinking level.

	Mean	SD	SEM	*t*	*df*	sig.
Control Q1–control Q2	−2.30	7.94	1.25	−1.83	39	0.075

**TABLE 4:** Descriptive statistics and *t*-tests results of both groups in the second administration of the Reflective Thinking (RT) Questionnaire.

Groups	*N*	Mean	SD	*t*	*df*	Sig
Experimental	40	59.55	5.25			
Control	40	55.67	6.06	3.05	78	0.00

In fact, Table 1 shows how both groups answered the questionnaire of the study for the first time.

Considering the above tables, it seems that the RT level of the experimental group has improved; the obtained value is 0.000, which significantly points to the difference between the first and the second performance of the participants who practiced reflective journal writing. The performance of both groups was then compared.

Regarding the means, it seems that the experimental group outperformed the control group on the second administration of the Reflective Thinking Questionnaire. The results of the independent sample *t*-test comparing the RT scores of journal writers and non-journal writers (Table 4) suggest that reflective journal practicing does have an effect on the reflective thinking (abbreviated as RT in the tables) levels of participants. Specifically, our results suggest that, when language learners practice reflection, they can enhance their RT level.

Table 4 indicates that *Sig* (0.00) is less than 0.05, meaning that the difference between both groups was significant at *p* < 0.05. Indeed, the experimental group outperformed the control group on the RT questionnaire.

### Structure of Journal Sentences

During the first four sessions, most of the sentences that participants presented were a simple (subject + verb + object) structure, but after session four, more complex structures were observed as participants used verbs like justify, classify, argue, and criticize, which showed higher planes of thinking (Bloom, 1956). That is, the learners could follow subject + verb + noun clause structure smoothly, which showed their writing skill enhancement. While monitoring the AR, the instructor found that the learners considered journaling as an option to keep track of their writing skill improvement. It seemed that almost all the learners’ writing skills improved after session four of the research.

### Class Achievement Results

To find out whether a significant difference exists between the experimental and control groups in terms of their final exams, a *t*-test was run. Descriptive statistics and *t*-test results are presented in Table 5.

**TABLE 5:** Descriptive statistics and *t*-test results of both groups’ performance on the final exam.

Groups	*N*	Mean	SD	*t*	*df*	Sig
Experimental	40	76.10	5.25			
Control	40	77.12	6.06	−0.50	78	0.61

In Table 5, the means of both groups are almost equal. The experimental group’s mean score is 76.10 and the control group’s mean score is 77.12. As Sig (0.61) is greater than 0.05, the difference between the groups is not significant at *p* < 0.05. In fact, they performed similarly on their final exam.

## Discussion

The results of the analysis indicated that most participants (36 out of 40) suggested reflective journals as positive and reported that reflective journals enhanced their awareness and understanding of learning. Interview results and analytical analyses also revealed that not only did learners extend their reflective practices to their learning and life skills, but they also developed their own level of RT. When learners probe into past experiences for new understanding, reflection is most likely to happen (Sargent, 2015). However, the first finding of our study is different from what Turhan and Kirkgoz (2018) suggested. They found that their EFL participants avoided giving any comments or insights in relation to what they observed and they solely reported what they observed in a descriptive way. Turhan and Kirkgoz (2018) added that individuals may tend to reflect descriptively in the absence of guidance or special training. Furthermore, through regular practices, reflection becomes a useful academic asset that helps students stabilize their knowledge and develop more sophisticated life skills and thoughts (Pretorius and Ford, 2016). Some studies have also reported that learners viewed reflective journal writing useful in developing awareness of their own learning (Zulfikar and Mujiburrahman, 2018). They also indicated that multiple means can highlight the importance of varied practices that help students demonstrate their learning (Rose and Meyer, 2002). Our findings also reported that individuals who practiced reflective journal writing outscored those who did not practice reflective journals. Monitoring one’s own learning encourages learners to have a more determining role in the process. Students can move from knowledge receivers to critical thinkers (Karimnia and Tahmasbi, 2017) as they are enabled to set their own goals in a learning situation and to check whether they have gained what they were looking for. Consequently, as Guthrie and Jones (2012) noted, intentional and prompted discussions about reflective experiences with peers and teacher can help learners better reflect on their own thoughts and understanding. Messiou (2018) found through the process of AR that teachers and students cultivated closer relations in their ways of working, as well as in their ways of understanding each other. That is, a change of practices resulted in moving toward practices that were more inclusive of all practices common to educational settings.

In line with McGarr and Moody (2010), at the onset of the present study, it was thought that the workload of the writing journals, through which students were required to reflect occasionally, could damage quality for quantity in the written reflections. Furthermore, due to problems that usually block journal writing (Soodmand Afshar and Farahani, 2018), students may be reluctant to develop reflection. However, as similar studies also found (Frazier and Eick, 2015), our findings confirmed Hume’s (2009) argument who agreed keeping a journal was considered time-consuming but “gratifying” by the participants. However, such initial doubts and concerns for learning may target what we, as teachers, material developers, and policymakers, have instilled in our students’ minds as learning. Mostly, in the Iranian classes learning means reading and reciting the memorized points. More teacher-student collaborative research is required to positively change such doubts toward alternative classroom actions.

Regarding question number two, the results of examining the structure and content of the learners’ reflective journals showed a considerable improvement in learners’ writing. The verbs utilized in the first four sessions of the research were mostly related to the first or second classification of Bloom’s Taxonomy of Questions (Bloom, 1956), like know, understand, and think. This finding can show reflective journal writing affected the learners’ style of writing and the use of verbs. Seemingly, after session four of the practice, the learners used the verbs of higher levels of thinking concerning Bloom’s (1956) Taxonomy. Moreover, the complex sentences in reflective journals were noticeable examples. Even at their interviews, participants agreed that their writing skills have improved while journal writing; they added they could make noun clauses and complex sentences easily and smoothly in their writing. Although the problems that Soodmand Afshar and Farahani (2018) mentioned are present, and despite what Otienoh (2009) argued that the participants in her study hardly engaged in journal writing since they were not exposed enough to the concept of reflective writing, our participants’ journals and interviews suggested that they could transfer reflection into their workplace. In their own terms, the participants defined the change as “thinking and behaving differently” and “greater confidence.” Griggs et al. (2018) also reported that students could transfer their reflective learning into the work context. Also similar to what similar investigations have suggested (e.g., Hume, 2009; Otienoh, 2009; Frazier and Eick, 2015), almost all the participants of the present study purported that reflective journal writing could lead them to pour out their feelings and reflection toward class life. Furthermore, connections between teachers and learners were tighter through the feedback provided to the learners, and through the reflections they wrote. Marzban and Ashraafi (2016), argued that improving reflection skills can enhance the psychological features of the instructor-learners’ reciprocal relationship which was met in the present study too. Although it is debated that it is difficult for high school students to apply reflective skills (White and Frederiksen, 2005; van Velzen, 2015), the findings of the present study may pave the way for reflecting for the participants by providing exemplars. In the interviews, the learners commented on the worth of exemplars for picturing how their reflective writing would be. Furthermore, they generally expressed their gratitude toward the teacher’s feedback, since they reckoned the feedback promoted them to think more deeply.

Concerning the third research question, the findings illustrated that class achievement may not be directly related to the learners’ level of RT hence different from similar previous studies. Bataineh and Zghoul (2006) highlighted that a higher class achievement had a positive relationship with applying RT skills. Soodmand Afshar and Farahani (2018), said in brief, that the success of students in their academic studies was attributed more to critical thinking than to learning strategies. Messiou (2018) found through the process of collaborative AR, teachers and students became closer in their ways of working, as well as in their ways of understanding one another. As a result, we saw a change of practices, moving toward practices that were more inclusive of all. Hence, no significant difference existed between the two groups in terms of their class achievement. A possible justification for these results is related to the nature of final exams. In fact, their final exam is a multiple-choice item test that is based on the course content. To perform well on such tests, students should read to do the test. In line with Bloom (1956), it can be said that, at the knowledge/remembering level, just memorization and recall are involved and the lower order cognitive skills are engaged. From a different point of view, it can be argued that both groups achieved the goals of the course. They all obtained scores and could pass the course. However, in usual language classes, learners are not expected to go further than recalling what is included in the course materials.

## Conclusion

Regarding AR, RT, and journal writing, there are views that are taken almost for granted in Education: AR is the process of studying a school situation to understand and improve the quality of the educative process (Johnson, 2012). Different methods of reflective practices can foster RT and life skills (Ramlal and Augustin, 2020). However, there is a need to implement AR and develop reflective habits to follow a systematic process and to benefit from bridging the gap between research and practice, particularly in contexts where this gap is deeper. The present study suggested that reflective journal writing can positively affect the learners’ writing skills and reflection ability. Students learned how to involve themselves in introspection, reflect on learning experiences, and write down their thoughts in journals. Possibly, in the long term, the practices implemented in the present study have the potential to develop students’ higher-order critical thinking and evaluative skills.

As Carter (2012) argues, AR and reflective writing follow the same process since both aim at highlighting immediate problems, planning, acting, evaluating, and contributing outcomes. The literature on reflective practices research also suggests that teachers are prone to provide theoretical knowledge about reflection and implement reflective journal writing in order to practically involve students in RT learning. Indeed, reflection is critical to learning and reflective writing is beneficial for challenging courses (Hume, 2009; Otienoh, 2009). The instructional condition assigned to a course influences the number of student reflections related to reflections of higher specificity levels (Mirriahi et al., 2018).

This piece of research was based on the application of well-known theories and strategies to develop learners’ reflective skills. However, outstandingly, we found participants engaged actively in the reflective practice in various cycles of AR have engaged in their own learning. Furthermore, reflective skills were achieved by providing focused opportunities. Participants were involved by following reflective writing exemplars and regular written and oral feedback from the instructor. As Bruno and Dell’Aversana (2017) have highlighted, such cultivated trust between students and teachers can be a facilitating factor for reflective practices.

## Limitations of the Study and Suggestions for Further Research

The present study faced a handful of limitations; it included female language learners and followed their RT development in language classes. The age of the participants was not considered as a factor in analyzing the data. Explanatory factors that may account for differences in reflective writing practices have been suggested by previous studies and includes issues like gender, time, journal content, and feedback (Yu and Chiu, 2019; Salahi and Farahian, 2021). Factors like age, gender, the field of study, feedback, students’ backgrounds, and topic and the interplay between a couple of such factors are suggested to be regarded in analyzing the content of reflective journals for future investigations.

## Data Availability Statement

The raw data supporting the conclusions of this article will be made available by the authors, without undue reservation.

## Author Contributions

All authors listed have made a substantial, direct, and intellectual contribution to the work, and approved it for publication.

## Conflict of Interest

The authors declare that the research was conducted in the absence of any commercial or financial relationships that could be construed as a potential conflict of interest.

## Publisher’s Note

All claims expressed in this article are solely those of the authors and do not necessarily represent those of their affiliated organizations, or those of the publisher, the editors and the reviewers. Any product that may be evaluated in this article, or claim that may be made by its manufacturer, is not guaranteed or endorsed by the publisher.
